# Nutritional Status, Selected Nutrients Intake, and Metabolic Disorders in Bariatric Surgery Patients

**DOI:** 10.3390/nu15112479

**Published:** 2023-05-26

**Authors:** Iwona Boniecka, Aneta Czerwonogrodzka-Senczyna, Anna Jeznach-Steinhagen, Krzysztof Paśnik, Dorota Szostak-Węgierek, Samir Zeair

**Affiliations:** 1 Department of Clinical Dietetics, Faculty of Health Sciences, Medical University of Warsaw, 01-445 Warsaw, Poland; aneta.czerwonogrodzka@wum.edu.pl (A.C.-S.); anna.jeznach-steinhagen@wum.edu.pl (A.J.-S.); dorota.szostak-wegierek@wum.edu.pl (D.S.-W.); 2 Department of General, Gastroenterological and Oncological Surgery, Collegium Medicum Nicolaus Copernicus University, 87-100 Torun, Poland; kpasnik@gmail.com; 3 Department of General, Vascular and Transplantation Surgery, Marie Curie Hospital, 71-455 Szczecin, Poland; samirz11@hotmail.com

**Keywords:** nutritional status, nutrients, fatty acids, bariatric surgery, obesity

## Abstract

Bariatric surgery is the most effective treatment for obesity and its complications. However, failure to adhere to dietary recommendations can result in both unsatisfactory weight loss and metabolic disorders. The aim of this study was to evaluate the effects of bariatric surgery on the anthropometric parameters and selected nutrient intake. A total of 12 months postoperatively, percent excess weight loss (%EWL) was significantly higher after laparoscopic Roux-en-Y gastric bypass (LRYGB) than laparoscopic sleeve gastrectomy (LSG) and laparoscopic adjustable gastric banding (LAGB) (93.78% vs. 56.13% and 55.65%, *p* < 0.001). The same was true for waist-to-hip ratio (WHR) (*p* = 0.017) and waist-to-height ratio (WHtR) changes (*p* = 0.022). There was a significant decrease in total cholesterol (TC) and low-density lipoprotein cholesterol (LDL-C) levels after RYGB. A significant decrease (*p* < 0.05) in daily intake was found for energy (4278.4 kcal vs. 1355.17 kcal), sucrose (122.23 g vs. 38.22 g), dietary fiber (30.90 g vs. 14.20 g), eicosapentaenoic fatty acid and docosahexaenoic acid (EPA+DHA) (142.46 mg vs. 52.90 mg) and % energy from fats (42.43% vs. 35.17%), saturated fatty acids (SAFAs) (19.96% vs. 14.11%) and alpha-linolenic fatty acid (ALA) (0.87% vs. 0.69%). Energy intake and energy % from fats positively correlated with body weight (BW), waist circumference (WC), WHR, and WHtR, and negatively with %EWL. The percentage of unsaturated fatty acids positively correlated with WC and WHR. Energy intake correlated positively with serum triglycerides (TGs) and energy % from fats and carbohydrates. Despite significant weight loss, the patient’s diet deviated from recommendations and may have contributed to metabolic disorders.

## 1. Introduction

The prevalence of obesity has been steadily increasing for many years. The worldwide obesity rate has nearly tripled since 1975. In 2016, more than 1.9 billion adults, 18 years and older, were overweight. Of these, over 650 million were obese. This means that 39% of adults aged 18 years and older were overweight, and 13% were obese [1]. WHO estimates that by 2025, approximately 167 million people will become less healthy because they are overweight or obese [2]. 

Excessive body weight is associated with about 200 serious health consequences, including type 2 diabetes, hypertension, cardiovascular diseases, sleep apnea syndrome, joint diseases, nonalcoholic fatty liver disease (NAFLD), hyperlipidemia, and female infertility [3]. Population-based studies show that for every one standard deviation increase in BMI, the odds of T2DM increase by 67% and those of coronary artery disease by 20% [4]. The conservative treatment of obesity, i.e., using diet and physical activity, is very difficult. Therefore, bariatric surgery has been gaining special recognition in recent years. The surgical treatment of obesity is more and more commonly called metabolic surgery, as it alleviates obesity complications. It is due to the fact that metabolic disorders associated with obesity improve much faster than if only treated by means of weight loss [5]. Based on 2019 recommendations in Poland, bariatric surgery is particularly recommended for patients with BMI > 40 kg/m^2^ (class III obesity) and BMI > 35 (class II obesity) with comorbidities [5]. The main mechanisms affecting weight reduction and resolution of metabolic disorders after surgical treatment of obesity are reduced gastric capacity, changes in the secretion of gastrointestinal hormones, and reduced intestinal absorption [5]. Currently, laparoscopic sleeve gastrectomy (LSG) and laparoscopic Roux-en-Y gastric bypass (LRYGB) are most commonly performed worldwide. The frequency of performing laparoscopic adjustable gastric banding (LAGB) is decreasing [6].

However, performing the operation alone is insufficient for effective and safe weight reduction. Therefore, the role of proper dietary management in the postoperative period is so important [6,7,8,9,10,11,12]. It is crucial in postoperative weight reduction, the prevention of food shortages and intolerances, and the treatment of diet-related diseases [6,7,8,9,10,11,12,13,14,15,16,17,18,19,20,21,22,23,24]. Notably, weight loss after bariatric surgery does not always translate into balanced nutrition, which may lead to a recurrence and exacerbation of diet-related diseases [13,14,15,16,17,18,19,20,21,22,23,24]. However, it needs to be noted that it is very difficult to balance a diet after bariatric surgery due to the reduced stomach capacity and reduced absorption of nutrients [6,7,8,9,10,11,12,13,14,15,16,17,18,19,20,21,22,23,24]. In addition to deficiencies of protein and some minerals and vitamins, the patient may also suffer from the inappropriate intake of fatty acids (monounsaturated and polyunsaturated), carbohydrates, including dietary fiber, and the incorrect share of individual macronutrients in the energy value of the diet. Unfortunately, most guidelines for patients after surgical treatment of obesity focus on the first 4–8 weeks after surgery and supplementation [6,24]. There are no long-term recommendations relating to energy and macronutrients, dietary fiber intake, or the proportion of fatty acids in the diet. Meanwhile, their supply contributes significantly to weight maintenance after surgery, lipid and glycemic disorders, and also cancer and fertility disorders. Such an analysis has not been a frequent subject of research so far. Of course, it is important to remember that bariatric procedures are not an ideal treatment method. They result in the resolution of obesity and its complications, but they can also promote nutritional deficiencies due to limited intestinal absorption, gastroesophageal reflux, reactive hypoglycemia, bone loss, and surgical complications.

The aim of this study was to evaluate changes in anthropometric parameters and intake of selected nutrients in patients 12 months after three types of bariatric surgery in the context of the risk of metabolic disorders.

## 2. Materials and Methods

### 2.1. Participants

A total of 70 patients aged 40.7 ± 12.1 including 49 women (70%) were surgically treated for obesity at the General, Oncological and Metabolic Surgery Clinic of the Military Institute of Medicine in Warsaw. Study participants included obese patients who underwent bariatric surgery for weight loss.

The follow-up period of the study was 12 months, and data were collected in two phases: preoperative (baseline, 1st stage of the study) and 12 months after the surgery (2nd stage of the study). 

Inclusion criteria were body mass index (BMI) in the range of 35–40 kg/m^2^ with obesity-related diseases in which weight loss may potentially improve (e.g., type 2 diabetes, hypertension, cardiovascular diseases, sleep apnea syndrome, joint diseases requiring surgery, nonalcoholic steatohepatitis (NASH), nonalcoholic fatty liver disease (NAFLD), hyperlipidemia, female infertility, also due to polycystic ovary syndrome) as well as BMI ≥ 40 kg/m^2^ with or without mentioned comorbidities [6].

The exclusion criteria were: 

Incurable diseases leading to cachexia (e.g., active cancer, acquired immunodeficiency syndrome (AIDS)); diseases that are life-threatening in the short term (e.g., fresh myocardial infarction); uncompensated endocrine diseases underlying obesity (e.g., Cushing’s syndrome); severe coagulation disorders; and lack of cooperation on the part of the patient or lack of acceptance of the effect of the procedure are caused by: active alcohol or drug addiction, mental illnesses uncontrollable despite treatment and pharmacotherapy, mental impairment of a severe degree; inability to participate in ongoing long-term follow-up after surgical treatment; the period of 12 months preceding the planned pregnancy, pregnancy, and lactation (until termination and completion of lactation); lack of consent from the patient, as well as lack of full conviction about the right choice of surgical treatment; conditions that prevent independent living when family or social care is unable to provide adequate long-term supervision [6].

All patients received a form informing them about the characteristics and the course of the study, and all of them signed an informed consent form agreeing to participate in the project. The study was approved by the Bioethical Commission of the Medical University of Warsaw (nos. KB/126/2006 and KB/66/A/2007).

### 2.2. Bariatric Surgical Procedures

The surgical interventions included laparoscopic adjustable gastric banding (LAGB)—27%, laparoscopic Roux-en-Y gastric bypass (LRYGB)—37%, and laparoscopic sleeve gastrectomy (LSG)—36%. The decision on the type of surgery was made jointly by the surgeon (after consulting the dietitian) and the patient.

LAGB consists in placing an adjustable silicone band at the level of the cardia, creating a small stomach pouch above the band, with the rest of the stomach below the band. The gastric band is connected by a silicone tube to a subcutaneous reservoir. The reservoir can be filles or emptied to control the opening between the pouch and the remainder of the stomach. The stomach capacity after LAGB is 15–50 mL. Even though this procedure is associated with the lowest short-term complication rate, it is associated with a high long-term complication rate and weight regain, which has led to its progressive replacement by sleeve gastrectomy. 

LRYGB involves the creation of a small gastric pouch (<50 mL volume), which is connected to the proximal small bowel by bypassing the first 75–150 cm and bringing a 100–150 cm alimentary limb onto the gastric pouch. This type of surgery results in reduced food intake, reduced nutrient absorption, as well as an increase in the secretion of intestinal hormones responsible for improving glucose metabolism 

LSG involves resection of the lateral part of the stomach to create a narrow gastric tube along the lesser curvature. The volume of the stomach after LSG is about 150 mL. It promotes weight loss through reduced gastric capacity and reduced appetite (decreased ghrelin secretion) [25]. 

### 2.3. Anthropometric Measurements

Body weight (BW) was measured with high-quality electronic calibrated scales and height with a wall-mounted stadiometer (to the nearest 0.5 cm). Body mass index (BMI) was calculated as weight in kilograms divided by the square of height in meters [26,27]. 

Weight loss outcomes after bariatric surgery were assessed using percent excess weight loss (%EWL) and percent excess BMI loss (%EBMIL) [28].

%EWL was calculated using the formula:

%EWL [(Initial Weight) − (Postop Weight)]:[(Initial Weight) − (Ideal Weight)] × 100

In which ideal weight was defined by the weight corresponding to the BMI of 25 kg/m^2^.

%EBMIL was calculated using the formula:

[∆ BMI/(Initial BMI − 25)] × 100

Changes in BMI (Δ BMI) were calculated using the formula [28]:

∆ BMI = (Initial BMI − Postop BMI)

To identify abdominal obesity, which increases the metabolic risk (morbidity and mortality), the following indicators were used: waist circumference (WC), waist-to-hip ratio (WHR), and waist-to-height ratio (WHtR). According to WHO recommendations [26,27,29], waist circumference (WC) was measured at the midpoint between the lower margin of the last palpable rib and the top of the iliac crest, using a stretch-resistant tape. Hip circumference was measured around the widest level of the buttocks, with the tape parallel to the floor. Each measurement was repeated twice. If the measurements were within 1 cm from one to another, the mean was calculated. If the difference between the two measurements exceeded 1 cm, the two measurements were repeated. The waist–hip ratio (WHR) was calculated by dividing waist circumference by hip circumference. According to the International Diabetes Federation for Europids and WHO, abdominal obesity was indicated with WC exceeding 80 cm in women and 95 cm in men [29]. According to WHO, abdominal obesity was defined as a waist–hip ratio above 0.90 for males and above 0.85 for females [29].

The waist-to-height ratio (WHtR) was calculated by dividing WC by height. WHtR ≥ 0.5 is considered to be linked to early health risk [30].

### 2.4. Blood Collection and Analysis

Blood samples were collected after an overnight fast (8–14 h after the previous meal) before the operation and 12 months later. Biochemical analyses were performed in the local laboratory. They included fasting plasma glucose (FPG), total cholesterol (TC), low-density lipoprotein cholesterol (LDL-C), high-density lipoprotein cholesterol (HDL-C), and triglycerides (TGs) concentrations. 

The following methods were used in the analysis of the blood concentration of the biochemical parameters: FPG—enzymatic method with hexokinase, TC and TG—enzymatic-colorimetric method, HDL-C and LDL-C—homogeneous colorimetric enzymatic method.

Blood test results were compared against the standards set out in the recommendations “Standardized Outcomes Reporting in Metabolic and Bariatric Surgery” [28].

### 2.5. Dietary Intake

Follow-up interviews were conducted during clinical appointments. During the clinical appointment, patients met a surgeon and a dietitian. The surgeon evaluated the patients before the surgery and their recovery after surgery. 

During consultations with a dietitian (before the surgery and 12 months after the surgery), anthropometric measurements were made, the 24 h dietary recall (interviews about consumption in the past 24 h) was analyzed, the patient’s current diet was corrected, and dietary advice was provided. Using the 24 h dietary recall, food consumption data were collected. Therefore, the patients completed a special questionnaire with details concerning all foods and beverages consumed on the previous day and the time of meals. During each meeting with a dietitian, the size of individual meals as specified by the patient after becoming familiar with the portion sizes included in the “Photo Album of Products and Dishes” [31]. To assess the energy and nutritional value of patients’ menus (obtained from the 24 h dietary recall), the Diet 5 software was used. 

The energy and nutritional value of the diet was calculated, including dietary energy intake (kcal), total protein (grams, % of the total calorie intake), carbohydrates (grams, % of the total calorie intake) including sucrose (grams, % of the total calorie intake) and dietary fiber (grams), fats (% of the total calorie intake), saturated fatty acids (SAFAs), monounsaturated fatty acids (MUFAs), polyunsaturated fatty acids (PUFAs) (% of the total calorie intake), linolenic fatty acid (LA), alpha-linolenic fatty acid (ALA), eicosapentaenoic fatty acid (EPA) and docosahexaenoic acid (DHA). The obtained results were compared to nutrition standards (Table 1). Due to the lack of uniform recommendations regarding nutrient intake standards after bariatric surgery, the results obtained were compared to the recommendations of the Clinical Practice Guidelines for the Perioperative Nutritional, Metabolic and Nonsurgical Support of the Bariatric Surgery Patient (the American Association of Clinical Endocrinologists (AACE), the Obesity Society (TOS), and the American Society for Metabolic & Bariatric Surgery (ASMBS)) [6], European Guidelines for Obesity Management in Adults [32], Clinical recommendations for the management of obese patients 2022—the position of the Polish Society for the Treatment of Obesity [33], The National Institute of Public Health—National Institute of Hygiene or NIPH–NIH [34], Diabetes Poland [35].

### 2.6. Statistical Analysis

Statistical analysis was performed with the use of descriptive statistics, the mean, median, minimum, and maximum values. The Kolmogorov–Smirnov test was used to verify the assumption of the normal distribution of variables. Statistically significant deviations from the normal distribution of blood glucose and triglyceride concentrations were reported in the case of biochemical tests and in the case of nutrient consumption and carbohydrate intake (g) in the percentage of energy intake from ALA, EPA, DHA (mg), and cholesterol (mg).

In the analysis of anthropometric parameters, the descriptive statistics were complemented with Student’s *t*-test values for dependent samples.

As regards the biochemical tests, the descriptive statistics were complemented with the values of a test of the statistical significance of differences. Indices whose distributions were similar to the normal one was analyzed with a Student’s *t*-test for dependent samples. The remaining indices were analyzed with the Wilcoxon test.

As regards the energy and nutritional value of the diet, the descriptive statistics were complemented with the values of a test of the statistical significance of differences.

The analysis of variance with repeated measurements in a mixed model was performed in order to verify whether the type of surgery was the factor that differentiated the course of changes between measurements during the 1st and 2nd stages in the case of normally distributed variables. Variables that were not normally distributed were analyzed with the non-parametric Wilcoxon test. 

The analysis of correlation was used to verify the presence of correlations between anthropometric and biochemical parameters and the consumption of energy and nutrients and correlations between biochemical parameters and the consumption of energy and nutrients.

Statistical analyses were performed with W SPSS 26.0 software with the level of statistical significance set at *p* < 0.05.

## 3. Results

### 3.1. Anthropometric Measurements

As shown in Table 2, during the observation period, statistically significant differences were found between the results of all anthropometric measurements (*p* = 0.001). %EWL and %EBMIL after 12 months were 69.99 ± 26.11%, and ΔBMI was 14.76 ± 4.82 kg/m^2^.

### 3.2. Anthropometric Parameters and Type of Bariatric Surgery

It was found that changes in anthropometric parameters (except WHR) 12 months postoperatively compared to the preoperative period depending on the type of bariatric surgery. Changes in BW and BMI in patients after LSG and LRYGB were greater than those obtained in the group after LAGB. The BW after LSG decreased from 156.63 ± 28.40 kg to 110.37 ± 19.90 kg, after LRYGB from 119.63 ± 16.71 to 75.43 ± 14.26, whereas after LAGB from 126.33 ± 14.64 kg to 92.99 ± 10.94 kg (*p* = 0.009). In the case of BMI, the respective values were from 53.00 ± 7.28 kg/m^2^ to 37.41 ± 5.76 kg/m^2^, from 42.40 ± 4.81 to 26.69 ± 4.15 and from 46.72 ± 4.94 kg/m^2^ to 34.36 ± 3.54 kg/m^2^ (*p* = 0.037). 

%EWL and %EBMIL were significantly higher (*p* < 0.001) after LRYGB (93.78 ± 22.46%) than LAGB (55.65 ± 16.93%) and LSG (56.13 ± 15.63%). ΔBMI did not depend on the type of surgery (15.72 ± 3.65 kg/m^2^, 12.36 ± 4.89 kg/m^2^, 15.58 ± 5.37 kg/m^2^, respectively, *p* > 0.05).

Changes in WC and WHtR obtained in the group after LRYGB were greater than those in the group after LSG and LAGB (*p* = 0.017 and *p* = 0.022, respectively). WC decreased from 120.04 ± 12.12 cm to 84.88 ± 10.08 cm (LRYGB), from 141.76 ± 19.28 cm to 110.28 ± 16.20 cm (LSG), and from 129.00 ± 8.22 cm to 102.21 ± 8.15 cm (LAGB). WHtR decreased from 0.72 ± 0.07 to 0.51 ± 0.05 after LRYGB, from 0.79 ± 0.06 to 0.62 ± 0.06 after LAGB, and from 0.82 ± 0.09 to 0.64 ± 0.08 after LSG.

### 3.3. Biochemical Blood Tests

According to Standardized Outcomes Reporting in Metabolic and Bariatric Surgery [28], preoperatively too-high FBG, TC, LDL-C, and TG levels were found in 58.9%, 68.5%, 56%, and 46.6% of patients, respectively, and too-low HDL-C levels in 49.3% of patients. One year after surgery, the percentage of patients with abnormal blood levels of these parameters statistically significantly (*p* < 0.0001) decreased (to 13.7%, 32.9%, 35.6%, 12.3%, and 8.2%, respectively). As shown in Table 3, significantly lower values of glucose, total cholesterol, triglycerides, and LDL cholesterol, as well as a statistically significant increase in HDL cholesterol, were obtained 12 months later compared to the postoperative period.

### 3.4. Biochemical Parameters and Anthropometric Parameters

Both before surgery and 12 months later, statistically significant correlations were found between the analyzed anthropometric and biochemical parameters. Before the operation, positive correlations were found for all analyzed anthropometric parameters and glucose. HDL-C correlated negatively with body weight, WC and WHR. TG concentration correlated positively with WHR.

As shown in Table 4, after 12 months, the concentration of FBG and TG correlated positively with BW, BMI, WC, WHR, and WHtR and negatively with %EWL, ΔBMI, and %EBMIL. TC and LDL-C correlated positively with BMI and WHtR but negatively with %EWL and %EBMIL. HDL-C concentration correlated negatively with BW, BMI, WC, WHR, and WHtR and positively with %EWL and %EBMIL.

### 3.5. Biochemical Parameters and Type of Bariatric Surgery

It was found that biochemical parameters differed depending on the type of bariatric surgery. Significant interaction effects were demonstrated between the type of surgery and changes in TC (*p* = 0.020) and LDL-C (*p* = 0.004) (Table 5). The changes occurred only for LRYGB. TC decreased from 197.88 ± 26.12 mg/dl to 165.19 ± 28.05 mg/dL. The respective values for LSG and LAGB decreased from 208.6 ± 45.68 mg/dL to 193.68 ± 39.38 mg/dL and from 204.68 ± 44.29 mg/dL to 195.58 ± 36.55 mg/dl. LDL-C decreased from 118.38 ± 21.09 mg/dL to 86.65 ± 25.93 mg/dL for LRYGB, from 125.48 ± 36.64 mg/dL to 118.60 ± 35.82 mg/dL for LSG, and from 117.21 ± 35.26 mg/dL to 106.53 ± 35.38 mg/dL for LAGB.

In the case of a decrease in FBG and TG, no dependence on the type of surgery was found.

### 3.6. Energy and Selected Nutrient Intake

One year after the operation, a significantly (*p* = 0.001) lower consumption of energy (kcal), proteins (g), carbohydrates (g), sucrose (g), dietary fiber (g), cholesterol (mg), and EPA and DHA acids (mg) was demonstrated (Table 6). In the case of EPA and DHA acids, due to a very large dispersion (min. 0.0 mg, max. 5380 mg) of the results, the median was much lower than the mean (43.00 mg vs. 142.46 mg before the operation and 9.50 mg vs. 52.90 mg 12 months later). Moreover, the percentage of the total calorie intake from fats (*p* = 0.001), the percentage of the total calorie intake from SAFA (*p* = 0.001), and the percentage of the total calorie intake from ALA (*p* = 0.014) were significantly lower. In turn, the percentage of the total calorie intake of proteins was significantly higher (*p* = 0.001).

### 3.7. Energy, Selected Nutrient Intake, and Anthropometric Parameters

It was shown that before the bariatric surgery, the higher the energy consumption, the higher the BW, BMI, WC, and WHR were (Table 7). Protein consumption (g) was positively correlated with BW, WC, WHR, and WHtR. The percentage of the total calorie intake of fat correlated positively with BW, BMI, WC, and WHtR, and the percentage of monounsaturated fatty acids in the energy value of the diet—with body weight, BMI, and WHtR. BW increased with carbohydrate consumption (g). However, when the percentage of the total calorie intake from sucrose increased, the values of BW, WC, and WHtR decreased. In the case of dietary fiber, the opposite relationship was demonstrated. The percentage of the total calorie intake from carbohydrates correlated negatively with BW, BMI, WC, and WHtR.

Twelve months after the operation, it was found that higher energy consumption and the percentage of the total calorie intake from fat translated into higher BW, WC, WHR, and WHtR values and lower %EWL, %EBMIL, and ΔBMI. With the exception of ΔBMI, similar relationships were found in the case of the percentage of the total calorie intake from MUFA. As regards the percentage of PUFA, ALA, LA acid, and cholesterol, positive correlations were noted for WC and WHR. Higher percentages of the total calorie intake from carbohydrates translated into higher %EWL, %EBMIL, ΔBMI, and lower WC, WHR, and WHtR values.

### 3.8. Energy, Selected Nutrient Intake, and Biochemical Parameters

An increased energy intake (kcal) and carbohydrates (g) were associated with lower HDL-C preoperatively. In case of proteins, a negative correlation was found with the concentration of HDL-C. The percentage of the total calorie intake from fat correlated positively with FBG. As regards the concentration of glucose, it correlated negatively with the consumption of sucrose (g), the percentage of the total calorie intake from sucrose and the percentage of the total calorie intake from carbohydrates (Table 8).

One year after the surgery, it was shown that the energy value of the patient’s diet and the percentage of the total calorie intake from fats, MUFA, and carbohydrates correlated positively with the concentration of triglycerides. The correlation was negative for the percentage of the total calorie intake from proteins. The percentage of the total calorie intake from ALA correlated positively with TC and LDL-C.

### 3.9. Energy, Selected Nutrient Intake, and Type of Bariatric Surgery

Significant interaction effects were demonstrated between the amount of sucrose consumed (*p* = 0.01), the percentage of sucrose in total calorie intake (*p* = 0.010), the percentage of carbohydrates in total calorie intake (*p* = 0.037), and the type of surgery. A total of 12 months postoperatively, the change in sucrose consumption was significantly greater in patients after LRYGB compared to other types of surgery. In patients who qualified for LRYGB, sucrose consumption was 168.24 ± 95.16 g. In the case of LSG, it was 92.71 ± 68.71 g, and for LAGB, it was 98.71 ± 51 g. After a year the respective values were 37.17 ± 14.56, 38.86 ± 19.40, and 38.80 ± 22.92. The percentage of sucrose in total calorie intake increased from 8.22 ± 5.94 to 11.29 ± 6.65 g after LSG and decreased from 15.26 ± 7.26 to 11.74 ± 4.42 g after LRYGB. No significant changes were reported after LABG (9.69 ± 3.93 vs. 11.02 ± 5.91). Changes concerning the percentage of carbohydrates in total calorie intake were more marked after LSG (from 39.44 ± 7.39% to 45.42 ± 7.84%). After LRYGB, a slight increase was noted from 46.45 ± 7.41% to 47.04 ± 5.29%, and after LAGB, the values decreased from 42.22 ± 6.88% to 41.44 ± 8.05%.

Twelve months postoperatively, significant interaction effects were also demonstrated in the case of the percentage of energy value from ALA and the level of EPA and DHA consumption. The percentage of ALA in total calorie intake was lower after LRYGB (0.83 ± 0.48% and 0.57 ± 0.24; *p* = 0.043). No statistically significant differences were observed in the remaining groups. After LSG, it was 0.94 ± 0.43 and 0.78 ± 0.41, and after LAGB, the respective values were 0.84 ± 0.34% and 0.72 ± 0.41%. 

The total level of EPA and DHA consumption was significantly lower in the group of patients after LSG (*p* = 0.015): 261.4 ± 1067.12 (Mdn = 40, min = 0 mg, max = 5380 mg) vs. 27.04 ± 64.90 (Mdn = 10 mg, min = 0.00 mg, max = 328.00 mg). After LRYG, it was 51.00 ± 45.68 (Mdn = 41.50 mg, min = 0.00 mg, max = 159.00 mg) vs. 38.12 ± 76.25 mg (Mdn = 9.0, min = 0.00 mg, max = 362.00 mg), and after LAGB, it was 111.11 ± 239.37 mg (Mdn=43, min = 0.00 mg, max = 1076 mg) vs. 107.16 ± 238.08 mg (Mdn = 9.00, min = 0.00 mg, max = 913.00).

## 4. Discussion

The present study showed that significant changes occurred as regards the analyzed anthropometric, biochemical, and dietary parameters after 12 postoperative months. 

Changes in body weight loss and BMI in patients after LRYGB and LSG were significantly more marked than after LAGB, while %EWL and %EBMIL were the highest after LRYGB. ΔBMI did not depend on the type of surgery (15.72 kg/m^2^, 15.58 kg/m^2^ after LSG, and 12.36 kg/m^2^ after LAGB). %EWL after LRYGB was 93.78%, whereas after LSG and LAGB, 56.13 % and 55.65%, respectively. Notably, %EWL > 50 was assumed as the success of surgical obesity treatment [36]. A systematic review and network meta-analysis of randomized controlled trials showed that both RYGB and SG patients achieved similar body weight loss (%EWL and Δ BMI), so the effectiveness of both operations was significantly higher than AGB [37]. The mean %EWL for RYGB, SG, and LAGB were 67.3%, 71.2%, and 40.6%, respectively, and the changes in BMI were 13.5 kg/m^2^ for RYGB, 14.4 kg/m^2^ for SG, and 10.6 kg/m^2^ for LAGB [37]. A randomized clinical trial comparing the efficacy of LRYGB and LSG surgery showed that the 7-year mean %EWL was slightly greater after LRYGB than LSG, and the difference was not statistically significant [38]. The majority of studies showed that after a year following LRYGB and LAGB, the value of %EWL was lower than in the present study [36,37,39,40] and was estimated at 67% and 40%, respectively [37,39]. Artero et al. [40] demonstrated even lower values of %EWL in individuals who had undergone LRYGB. One year after surgery, it was 58.3%, Dinitz et al. [36] also reported that a year after LRYGB surgery, the value of %EWL was similar to the results of other cited studies (67.4%). In the case of LSG surgeries, the values of %EWL obtained by other authors were diversified and ranged from 57.7% to 71.2% [37,40,41,42].

A surprisingly suitable result achieved in the present research in the LRYGB group (>90%) might be due to the effect of the lower baseline body weight and BMI of those patients, which contributed to the fact that the average BMI was close to reference values after a year. Moreover, it was demonstrated [43] that lower BMI was noted in patients who had lost more weight. Patients with a baseline BMI ranging from 30 to 39.9 kg/m^2^ achieved 85.9 %EWL after a year post-RYGB. Similarly, Uno et al. [41] demonstrated that patients from a group characterized by lower baseline body weight (BMI 35–50 kg/m^2^) achieved higher %EWL values (96.3% after a year postoperatively) than patients with a BMI of 50–70 kg/m^2^ (73.4% after a year postoperatively). In this case, %EWL after a year was also higher in patients post-RYGB than post-LSG.

The present study showed that the baseline BMI of patients qualified for LRYGB was not significantly different from the BMI of patients qualified for LAGB. This, as well as the results discussed above, confirmed that LAGB was the least effective in the operative treatment of obesity, which was also reported by other authors [41,43]. 

The reduction in waist circumference and WHtR was significantly higher after LRYGB than LAGB and LSG, which may indicate a more beneficial effect of LRYGB surgery on the components of metabolic syndrome. Bettencourt-Silva et al. [39] demonstrated that two years after the surgery, WHtR was significantly higher in patients’ post-SG than post-LAGB. A study by Carvajal et al. [44] showed that normal WHtR was only achieved after 6 months post-LSG. The present study revealed that WHtR close to reference values was only achieved in post-LRYGB patients. However, it needs to be emphasized that the BMI of our patients was the lowest post-LRYGB, while according to Carvajal et al. [44], it was the lowest post-LSG. There was no correlation between WHR and LDL-C, while the correlation with WHtR was positive. This may confirm that WHtR may be a more sensitive indicator of cardiometabolic risk than the commonly used BMI, WC, or WHR [30,45,46]. Other authors also confirmed a higher usefulness of WHtR than WHR in the assessment of the risk of obesity complications [47]. Therefore, it seems that this indicator should be taken into account more often in the assessment of metabolic risk. A study concerning the influence of obesity on the pulmonary function in candidates for surgical obesity treatment showed that all the anthropometric parameters of obesity (BW, BMI, WC, WHtR) except WHR were associated with impaired pulmonary function [47].

The intake of the majority of analyzed dietary parameters significantly decreased during the observation period. Energy consumption after 12 months postoperatively decreased over 3-fold, which was higher than values reported by other authors [17,18,22,48,49] and similar to the results obtained by Coluzzi et al. [50]. It was about 1300 kcal and was consistent with the results of other studies [10,16,17,48,51,52]. However, according to Tabesh et al. [11], the intake during the first postoperative year did not exceed 1000 kcal/day. According to the latest recommendations, several months after surgery, patients should consume 1200 to 1500 kcal/day, with most patients consuming about 1500 to 1800 kcal/day, 6 months after surgery and long-term [5]. Several years of observations show that the range of energy value of the postoperative diet is variable and quite wide. It varies in the range of 900–2425 kcal per day [18,20,21,49]. The standards for calculating energy requirements which optimized the process of body weight reduction were not specified [14,28]. According to Via and Mechanick [53], indirect calorimetry should be a gold standard. However, it is currently hardly available. In such a situation, a specific mathematical formula may be used. However, the results of such calculations carry a risk of errors. Zarshenas et al. [18] emphasized that energy intake should depend on age, sex, and level of physical activity.

The postoperative stage revealed no differences in energy intake depending on the type of surgery, which was also confirmed by other authors [21,49,54], even in the course of a follow-up lasting several years [49]. The present study demonstrated that higher energy consumption was characterized by a positive correlation with body weight, waist circumference, WHR, and WHtR, and a negative correlation with %EWL and %EBMIL. The positive correlation between body weight and energy consumption was also confirmed by other authors [49,54]. Another study showed no correlation between energy intake and body weight increase during a 5-year follow-up. Patients whose body weight did not increase were characterized by a better-balanced diet with a higher protein and fat content. In such a case, the authors emphasized the significant role of other factors in the increase in body weight, including physical activity, poor dietary habits, lack of knowledge about nutrition, or eating disorders (bulimia) [20]. 

Regardless of the stage of the study, the intake of protein was within normal limits, although also in that case, it changed throughout the observation period. The percentage of protein increased (from 14.54% to 19.20%), and its consumption (in grams) diminished while satisfying the requirements related to the surgery (62.79 g/day). Such results were confirmed by other authors [17,50,51]. It was demonstrated that the percentage of protein in the energy value of the diet in patients after bariatric surgeries was in a wide range between 14% and 27% [10,17,18,20,21,48,49,50,51,54,55], with the average value amounting to 19–25% during the first postoperative year [17,18,48,51]. As regards the prevention of weight gain after LRYGB surgeries, Faria et al. [56] recommended an even higher percentage of protein—approx. 35% of the energy value of the diet. The Polish Diabetes Association [35] also permits a higher percentage of proteins in a low-energy diet, i.e., a targeted diet to which the patients should adhere. The results are also diversified if it comes to the analysis of protein intake in grams. A study conducted a year after the surgery showed that patients consumed from 46 to 96 g of proteins daily [55,57,58]. Longer (2–5 years) follow-ups demonstrated that protein consumption ranged from 49.7 to 77.5 g/day [16,20,21,50,58]. 

Proteins play a very important role, especially following bariatric surgery, as they assist in the healing of the postoperative wound [10,58,59], influence immunity [55,58], and prevent the use of the muscular tissue for energy [14,55,58]. It protects the patient from insulin resistance (because muscles play a role in glucose metabolism) and the reduction of the basal metabolic rate and, consequently, an increase in body weight [11,22,23,54,55,58]. Moreover, high-protein foods intensify the feeling of satiety, which also promotes maintaining normal body weight [10,11,14,55]. The ability of proteins to induce satiety could also be related to the levels of free amino acids in the blood, as these levels may signal satiety and lead to decreased food intake. What is more, amino acids are precursors to some neurotransmitters involved in regulating food intake, e.g., tryptophan is a precursor to serotonin. It has been noted that adequate protein intake can also affect the secretion of the satiety hormone PYY in the gastrointestinal tract [59]. The consumption of appropriate amounts of proteins is difficult after bariatric surgery due to the lower intake of food, reduced absorption related to the diminished production of hydrochloric acid and digestive enzymes [11,59,60], or the intolerance of high-protein products (especially meat) [9,11,14,55,59,60]. Food intolerance may sometimes persist for many years [9,11,14,55,59,60,61]. In addition, vomiting, diarrhea, depression, and alcohol consumption, can lead to or exacerbate protein malnutrition [59]. According to other studies, the minimal protein requirement, i.e., 60 g/day, was observed only in 46–64% of patients 12 months postoperatively [10,55,57,58].

Therefore, protein supplementation is recommended (approx. 30 g/day) [8,9,62]. Protein supplements are a suitable option because they have the advantage of increasing protein intake without increasing fat and carbohydrates [59]. It was demonstrated that it contributed not only to more marked weight reduction and the increase in fat-free mass index [62] but also to a more marked adipose tissue loss [63]. However, Schollenberg et al. [62] emphasized that the achievement of such results was possible if a diet included suitable protein intake. Their research showed that the largest percentage of recommended protein intake was covered by dietary proteins [63], so it seems that supplementation is not always necessary [50]. 

The percentage of carbohydrates in the energy value of the diet remained unchanged (42.80% preoperatively and 44.86% one year after surgery), and the intake was consistent with the results obtained by other authors [16,17,20,48,49,50,51,52,58]. A systematic review by Zarshenas et al. [18] revealed that the percentage of carbohydrates in the energy value of the diet in patients after bariatric surgeries was 38–52% (average: 40–45%). Although no consistent guidelines are available as regards the consumption of carbohydrates, it is most commonly recommended that they should account for 40–45% of the energy value of the diet [6,11,14,56] and come from minimally processed products with low glycemic load [56,64]. According to a 10-year follow-up, a higher intake may be associated with the risk of weight gain [23]. The present study showed that the percentage of carbohydrates in the energy value of the diet diminished to the largest extent following LSG. Such patients were also characterized by the highest body weight. The result may confirm a considerable percentage of carbohydrates in body weight gain [23,56,64].

The consumption of sucrose markedly decreased during the follow-up period (from 122.39 to 38.22 g/day), especially in post-LRYGB patients (from 168.24 to 37.17 g/day). What is more, after LRYGB, the percentage of fructose in total calorie intake decreased (15.26% vs. 11.74%). Such a result was confirmed by El Labban et al. [52], which may indicate the observance of recommendations concerning the reduction of the consumption of sugar and sweets in order to prevent dumping syndrome [14,23,52,56]. Miller et al. [17] demonstrated that 12 months after LRYGB surgery, sucrose consumption was higher than in the present study and amounted to 51 g, which corresponded with over 15% of the energy value of the diet. Notably, the intake of energy from sucrose was not commonly studied. Considering the significance of simple carbohydrates in body weight gain and the occurrence of dumping syndrome, the issue requires further analysis. 

Regrettably, at the same time, the consumption of dietary fiber decreased. The result is reflected in other research conducted by Moize et al. [49]. Other authors demonstrated an even lower consumption of fiber [17,18,20,50,64]. No explicit guidelines are available as regards fiber intake after bariatric surgery. However, according to Moize et al., the value should be about 14 g/1000 kcal in bariatric patients, which is consistent with the recommendations issued by the Polish Diabetes Association. Regrettably, regarding the fact of such a low energy value of the patient’s diet, the amount may be insufficient. The majority of recommendations estimate the consumption of 25–40 g of fiber. Therefore, Faria et al. [56] claimed that the supplementation of 15 g/day might be indicated. It is a well-tolerated amount. It is effective and recommended in the management of plasma lipid parameters (e.g., LDL-C) [65]. Regrettably, products rich in fiber (e.g., raw vegetables and legumes) may not be well-tolerated in bariatric patients [14], and the implementation of those recommendations may be hindered. 

Regardless of the phase of the study, the percentage of fats in the energy value of the diet was in the upper limit of the normal reference range [6,35] with the predominance of SAFA, which was considerably lower but still exceeded the normal limit by 70% preoperatively, and 40% a year later. Fats provided 42.43% of energy before surgery and 35.49% a year later. For SAFA, it was 19.96% and 14.11%, respectively. A similar percentage of fats in the energy value of the diet was reported both in bariatric patients [14,16,17,18,48,49,50,51,52,54,58] and in patients with metabolic syndrome who did not undergo surgery [65]. Although quite a high percentage of total fat consumption is acceptable, it needs to be emphasized that the intake of around 35–40% of the energy value of the diet is related to increased SAFA and energy consumption [66,67]. Therefore, it is indicated to maintain suitable proportions of the diet as regards individual fatty acids [65]. The predominance of SAFA, with the sources being mainly animal-origin fats, increases the risk of circulatory diseases, as SAFA is the most important diet-related factor of LDL-C and TC increase [65,66,67]. MUFA and PUFA may replace SAFA in the diet to prevent atherosclerosis and cardiac diseases [66,67]. The present study showed the insufficient consumption of MUFA (<20% of the daily energy requirement). PUFA, both preoperatively and 12 months later, was similar to the recommendations, i.e., about 6–10% of energy. Similar results were obtained by other authors [17]. Moize et al. [49] reported that the percentage of individual groups of fatty acids in the energy value of the diet was similar to the recommendations except for PUFA. However, the study was conducted in the Mediterranean population. A study by Verger et al. [58] showed that the insufficient intake of PUFA in the energy value of the diet was noted in RYGB patients, while in SG patients, it was similar to the one in the present study. Lim et al. [68] emphasized favorable changes that occurred in the nutrition of a 51-year-old patient 6 months post-LSG (SAFA intake reduction, the increased intake of MUFA and PUFA).

The present study showed that %LA (ω-6 group PUFA) and %ALA (ω-3 group PUFA) were approximating the reference ranges regardless of the phase of the study. In the case of %ALA the ranges were even exceeded. However, the consumption of other ω-3 group acids, i.e., EPA and DHA, was insufficient (57% and 20% of the reference values, respectively) compared to the requirements (250 mg/d), which might increase the risk of circulatory system diseases in the study group. PUFAs (ω-3 and ω-6) need to be provided with food, as they are not synthesized by the body [24,69,70,71]. It is known that, depending on the dose, the regular consumption of ALA is a substrate for the production of EPA and DHA, which present a multidirectional positive potential in the body [69,70,71]. Moreover, it was demonstrated that the total consumption of PUFA, LA, and ALA was negatively correlated with abdominal obesity and hypertension [65]. Another study showed that the preoperative level of LA in the blood was a predictive factor of BMI reduction, which might be suggestive of its favorable influence on weight loss [72]. ALA is a substrate for the production of EPA and DHA, which present a multidirectional positive potential in the body [69,70,71]. They demonstrate the following properties: neuroprotective, anti-depressive, anti-inflammatory [34,69,70,71,72,73], hypotensive, anti-thrombotic, and hypolipidemic (mainly diminishing plasma TGs, suppressing their synthesis in the intestinal wall and liver, and increasing their disintegration in the process of β-oxidation in the mitochondria) [69,70,71]. Additionally, a correlation was confirmed between low EPA concentrations and vitamin A, hyperinsulinemia, and high CRP, which might increase the risk of insulin resistance and aggravate obesity-related inflammation. EPA also plays an important role in the occurrence of depression, which is frequently associated with obesity [72,73].

The present results concerning the intake of EPA and DHA were corroborated by a systematic review that showed that PUFAs were consumed in amounts consistent with recommendations in only 26% of European countries. The recommended consumption of LA was reported in 52% of countries and of ALA in 77% of countries [74]. Forbes et al. [24] demonstrated that RYGB was associated with alterations in essential fatty acids within plasma phospholipids, including decreases in precursors and intermediary fatty acids (LAs and EPAs). It was probably related to the diminished consumption and absorption of fats, but it might also increase DHA concentrations, which the authors explained by increased biosynthesis. Hierons et al. identified reductions in the plasma concentrations of saturated, monounsaturated, and polyunsaturated fatty acids [19].

The present study showed a significant reduction in dietary cholesterol intake, which exceeded the reference values by over 2-fold before surgery and was within normal limits 12 months after surgery. Other authors also emphasized a considerable decrease in cholesterol consumption [17,68]. The intake of cholesterol should be limited mainly in individuals with its high levels in the plasma [35].

Due to limited food intake, dietary errors, and reduced absorption, bariatric surgeries may lead to a deficiency of omega-3 fatty acids. Their release from the adipose tissue in the process of body weight reduction is not always sufficient and optimal from the viewpoint of nutrition because of the limited storage capacity of the adipose tissue as regards omega fatty acids [75]. The present paper demonstrated a marked reduction in the consumption of EPA and DHA following LSG and a decrease in %ALA after LSG and LRYGB. Lin et al. [75] compared the concentrations of fatty acids in the blood before bariatric surgery and 12 months later. They demonstrated that in the case of LSG surgeries, the concentrations of fatty acids initially decreased, and after a year postoperatively, they returned to the preoperative value, except EPA acid. However, after BPDS (biliopancreatic diversion with a duodenal switch) surgeries, the majority of fatty acids remain at an insufficient level, which results from the reduced absorption of fats. Moreover, the preoperative results were compared to the control group, which revealed significantly lower omega-3 concentrations (ALA, EPA, DHA) in patients with severe obesity. It explained their increased use due to oxidative stress accompanying obesity. The authors suggested that omega-3 supplementation should be routinely recommended after bariatric surgeries until body weight normalization. Patients with EPA deficiency should be monitored in terms of cardiovascular pathologies.

The present study demonstrated a correlation between lipid and fatty acid consumption and anthropometric and biochemical parameters. The percentage of the energy value obtained from fats, regardless of the phase of the study, was positively correlated with body weight, waist circumference, and WHtR. Additionally, such a correlation was additionally observed for BMI before surgery and 12 months postoperatively with WHR. Surprisingly, %MUFA was also positively correlated with body weight and WHtR, and 12 months after surgery, also with WHR and waist circumference. One year after surgery, %PUFA, LA, and ALA were positively correlated with waist circumference and WHR. This may be related to the higher total percentage of fats in patients’ diets, which was confirmed in another study conducted on subjects with metabolic syndrome [65]. Preoperatively, such results should not be surprising, unlike the results of measurements taken 12 months postoperatively, especially with the significant reduction in energy consumption at that time. The cited study [65] also confirmed that the reduction in the intake of energy was accompanied by a higher percentage of dietary PUFA, MUFA, SAFA, LA, and ALA. Moreover, the result seems to corroborate opinions that WHtR is a more sensitive index of abdominal obesity than WHR, BMI, or even waist circumference. Furthermore, it is difficult to perform precise measurements of waist circumference, and the proportions of waist-to-hip circumference may be imprecise in patients with severe obesity. The percentage of energy obtained from fats and %MUFA were negatively correlated with the parameters of body weight loss (e.g., %EWL). It confirmed the significant percentage of fats in body weight increase and the fact that the higher total fat intake might be associated with higher consumption of unsaturated fatty acids. 

Based on a 10-year follow-up, Kanerva et al. [23] demonstrated that postoperative body weight reduction and its maintenance might be achieved by adherence to a diet in which proteins constituted about 20% of the energy value, fats < 35%, and carbohydrates >45%. A lower percentage of carbohydrates was necessary for the prevention of dumping syndrome. Therefore, the authors recommended a higher percentage of complex carbohydrates in the diet and the avoidance of simple carbohydrates. For that reason, Faria et al. [56] recommended that patients after LRYGB should consume slightly different proportions. Proteins should deliver 35% of the energy value of the diet, fats 20%, and carbohydrates—45% of the energy value of the diet. 

Limitations: 24 h diet recall, non-randomized study, short observation period.

## 5. Conclusions

One year after bariatric surgery, there was a significant decrease in the anthropometric and biochemical parameters analyzed, as well as changes in energy and nutrient intake. RYGB contributed to greater changes in anthropometric parameters and TC and LDL-C concentration. One year after surgery, however, fatty acids proportions were incorrect (with too-high SAFA and too-low EPA and DHA intake), and dietary fiber consumption was insufficient. Higher energy intake and a higher percentage of total calorie intake from fat after surgery were associated with lower weight loss and higher WHR and WHtR. Additionally, higher energy consumption and the percentage of total calories from fats and carbohydrate intake were associated with higher TG concentrations. 

Despite significant weight loss, the patient’s diet deviated from recommendations and may have contributed to metabolic disorders.

There is a need for further studies over a longer follow-up period and in larger groups to evaluate the effect of dietary patterns of patients undergoing bariatric surgery on metabolic risks.

## Figures and Tables

**Table 1:** Standards of energy and nutritional value of the diet.

Energy and Nutritional Value	Reference Values	References
Energy	≥1200 kcal/day	European Guidelines for Obesity Management in Adults [32], AACE/TOS/ASMBS [6]
Proteins	15–30% of the total calorie intake/day 60–80 g/day	Diabetes Poland [35] AACE/TOS/ASMBS/EASO/OMTF [6]
Fats	25–40% of the total calorie intake/day	Diabetes Poland [35]
Saturated fats (SAFAs)	<10% of the total calorie intake/day	Diabetes Poland [35]
Monounsaturated fats (MUFAs)	>20% of the total calorie intake/day	Diabetes Poland [35]
Polyunsaturated fats (PUFAs) Linolenic acid (LA) α-Linolenic acid (ALA) Eicosapentaenoic acid + Docosahexaenoic acid (EPA + DHA)	6–10% of the total calorie intake/24 h 4% of the total calorie Intake/day 0.5% of the total calorie intake/day 250 mg/day	Diabetes Poland [35] Standards of Nutrition for the Polish Population (The National Institute of Public Health—National Institute of Hygiene or NIPH–NIH) [34]
Cholesterol	<300 mg/day	Diabetes Poland [35]
Carbohydrates	45–60% of the total calorie intake/day	Diabetes Poland [35]
Sucrose	<10% of the total calorie intake/day	Diabetes Poland [35]
Dietary fiber	Min. 25 g/day or 15 g/1000 kcal	Diabetes Poland [35]

**Table 2:** Changes in anthropometric parameters across study duration.

Anthropometric Parameters	Stage of the Study	M	Mdn	SD	Min	Max	*t*	df	*p*
BW	1st	134.66	127.75	26.77	88.00	204.00	23.6	69	0.001
	2nd	92.68	90.75	21.63	51.10	143.30			
BMI (kg/m^2^)	1st	47.36	46.35	7.35	35.25	66.29	25.59	69	0.001
	2nd	32.60	33.22	6.59	19.00	52.64			
WC (cm)	1st	130.23	129.50	16.97	98.00	177.00	26.85	69	0.001
	2nd	98.66	98.50	16.41	66.00	138.00			
WHR	1st	0.93	0.93	0.09	0.73	1.18	8.47	69	0.001
	2nd	0.89	0.87	0.09	0.66	1.08			
WHtR	1st	0.77	0.77	0.09	0.62	0.98	27.19	69	0.001
	2nd	0.59	0.59	0.09	0.40	0.77			

1st—1st stage of the study (preoperative, baseline); 2nd—2nd stage of the study (12 months after the surgery); M—mean; Mdn—median; SD—standard deviation; min—minimum value; max—maximum value; *t*—Student’s *t*-test for independent samples; df—degrees of freedom; *p*—statistical significance.

**Table 3:** Changes in biochemical parameters across study duration.

Biochemical Parameters	Stage of the Study	M	Mdn	SD	Min	Max	*t*/*Z*	df	*p*	*d*
FBG (mg/dL)	1st	117.61	105.50	40.77	78.00	316.00				
	2nd	91.77	88.00	16.05	68.00	174.00	−6.75	-	0.001	0.73
TC (mg/dL)	1st	203.56	205.50	38.81	126.00	340.00				
	2nd	183.61	180.50	37.11	94.00	307.00	5.47	69	0.001	0.65
TG (mg/dL)	1st	161.31	144.50	72.88	63.00	355.00				
	2nd	102.27	86.50	61.68	36.00	423.00	−6.03	-	0.001	0.76
HDL-C (mg/dL)	1st	46.64	45.00	11.60	28.00	74.00				
	2nd	60.58	57.00	14.40	34.90	99.00	−9.38	69	0.001	1.12
LDL-C (mg/dL)	1st	120.60	120.50	31.08	56.00	212.00				
	2nd	103.46	104.00	34.76	31.00	203.00	4.90	69	0.001	0.59

1st—1st stage of the study (preoperative, baseline); 2nd—2nd stage of the study (12 months after the surgery); M—mean; Mdn—median; SD—standard deviation; min—minimum value; max—maximum value; *t*—Student’s *t*-test for dependent samples; *Z*—Wilcoxon’s test value; df—degrees of freedom; *p*—statistical significance; *d*—Cohen’s d effect size.

**Table 4:** Correlations between biochemical parameters and anthropometric parameters across study duration.

		Biochemical Parameters				
Anthropometric Parameters	Stage of the Study	FBG (mg/dL)	TC (mg/dL)	TG (mg/dL)	HDL-C (mg/dL)	LDL-C (mg/dL)
BW (kg)	1st	0.312 **	0.031	0.184	−0.312 **	0.070
	2nd	0.492 **	0.110	0.489 **	−0.367 **	0.182
BMI (kg/m^2^)	1st	0.391 **	0.084	0.078	-0.146	0.096
	2nd	0.471 **	0.257 *	0.497 **	−0.280 *	0.301 *
WC (cm)	1st	0.494 **	0.048	0.144	−0.253 *	0.051
	2nd	0.562 **	0.174	0.538 **	−0.370 **	0.208
WHR	1st	0.251 *	−0.012	0.244 *	−0.297 *	−0.024
	2nd	0.486 **	0.090	0.432 **	−0.303 *	0.079
WHtR	1st	0.541 **	0.081	0.059	-0.107	0.053
	2nd	0.548 **	0.271 *	0.526 **	−0.299 *	0.279 *
EWL	2nd	−0.547 **	−0.282 *	−0.581 **	0.269 *	−0.267 *
ΔBMI	2nd	−0.443 **	−0.088	−0.392 **	0.031	0.045
%EBMIL	2nd	−0.547 **	−0.282 *	−0.581 **	0.269*	−0.267 *

1st—1st stage of the study (preoperative, baseline); 2nd—2nd stage of the study (12 months after the surgery); * *p* < 0.05; ** *p* < 0.01.

**Table 5:** The values of interaction tests concerning the type of surgery as a moderator, the relationship between changes in the concentration of biochemical parameters and the type of bariatric surgery.

Biochemical Parameters	F	df	*p*	η^2^
TC (mg/dL)	4.15	2.67	0.020	0.11
HDL-C (mg/dL)	1.13	2.67	0.330	0.03
LDL-C (mg/dL)	5.97	2.67	0.004	0.15

F—test value; df—degrees of freedom; *p*—statistical significance, η^2^ (eta-squared)—effect size.

**Table 6:** Changes in energy and chosen nutrient intake across the study duration.

Nutrients	Stage of the Study	M	Mdn	SD	Min	Max	*t*/*Z*	df	*p*
Energy (kcal/)	1st	4278.40	3939.0	1287.81	2304.00	9061.00	20.03	69	0.001
	2nd	1355.17	1296.50	328.07	737.00	2627.00			
Proteins (g)	1st	150.73	140.50	47.84	66.80	341.90	15.49	69	0.001
	2nd	62.79	61.00	17.89	29.10	136.80			
Proteins (% of the total calorie intake)	1st	14.54	14.60	2.91	8.40	24.20	−7.15	69	0.001
	2nd	19.20	18.95	4.80	10.40	32.00			
Fats (% of the total calorie intake)	1st	42.43	42.20	6.51	27.10	56.30	6.47	69	0.001
	2nd	35.49	34.15	7.30	21.90	54.40			
SAFA (% of the total calorie intake)	1st	19.96	16.38	3.57	10.03	30.90	5.55	69	0.001
	2nd	14.11	13.72	3.53	6.93	23.68			
MUFA (% of the total calorie intake)	1st	17.54	17.43	3.74	9.31	25.61	1.12	69	0.268
	2nd	16.56	14.73	6.94	7.18	38.36			
PUFA (% of the total calorie intake)	1st	5.91	6.04	1.96	1.82	9.66	1.67	69	0.100
	2nd	5.31	4.77	2.41	1.78	11.14			
LA (% of the total calorie intake)	1st	4.95	5.01	1.69	1.52	8.76	1.30	69	0.197
	2nd	4.52	4.04	2.18	1.37	10.70			
ALA (% of the total calorie intake)	1st	0.87	0.83	0.43	0.22	1.96	−2.46	-	0.014
	2nd	0.69	0.55	0.36	0.27	1.78			
EPA + DHA (mg)	1st	142.46	43.00	648.33	0.00	5380.00	−2.95	-	0.003
	2nd	52.90	9.50	139.62	0.00	913.00			
Carbohydrates (g)	1st	485.30	475.05	164.88	125.90	863.70	−7.26	-	0.001
	2nd	163.15	164.25	40.74	64.70	268.00			
Sucrose (g)	1st	122.39	102.00	82.87	8.57	362.17	8.29	69	0.001
	2nd	38.22	38.32	18.58	1.42	85.22			
Sucrose (% of the total calorie intake)	1st	11.23	10.28	6.44	1.09	28.95	−0.16	69	0.876
	2nd	11.38	11.86	5.23	0.60	24.92			
Dietary fiber (g)	1st	30.90	28.90	10.53	13.60	57.80	13.02	69	0.001
	2nd	14.20	13.50	5.36	4.60	32.20			
Carbohydrates (% of total energy intake)	1st	42.80	43.45	7.77	19.00	62.30	−1.75	69	0.085
	2nd	44.86	45.50	7.36	21.90	64.70			
Cholesterol (mg)	1st	664.30	593.00	363.08	294.30	2748.30	-	-	0.001
	2nd	214.03	174.15	117.39	62.30	583.90			

1st—1st stage of the study (preoperative, baseline); 2nd—2nd stage of the study (12 months after the surgery); M—mean; Mdn—median; SD—standard deviation; min—minimum value; max—maximum value; *t*—Student’s *t*-test for dependent samples; *Z*—Wilcoxon’s test value; df—degrees of freedom; *p*—statistical significance.

**Table 7:** Correlations between energy, selected nutrient intake, and anthropometric parameters across study duration.

Energy/ Nutrients	Stage of the Study	Anthropometric Parameters							
		BW	BMI	%EWL	ΔBMI	%EBMIL	WC	WHR	WHtR
Energy (kcal)	1st	0.456 **	0.267 *				0.382 **	0.252 *	0.220
	2nd	0.313 **	0.233	−0.307 **	−0.253*	−0.307 **	0.391 **	0.375 **	0.335 **
Proteins (g)	1st	0.399 **	0.212				0.409 **	0.329 **	0.265 *
	2nd	0.087	0.026	−0.056	−0.099	−0.056	0.140	0.150	0.102
Proteins (% of the total calorie intake)	1st	−0.062	−0.072				0.075	0.144	0.094
	2nd	−0.199	−0.197	0.212	0.099	0.212	-0.210	−0.166	−0.203
Fats (% of the total calorie intake)	1st	0.317 **	0.390 **				0.256 *	−0.089	0.286 *
	2nd	0.320 **	0.225	−0.349 **	−0.337 **	−0.349 **	0.467 **	0.528 **	0.410 **
SAFA (% of the total calorie intake)	1st	0.185	0.191				0.155	−0.136	0.152
	2nd	0.026	0.093	−0.128	-0.154	-0.128	0.101	0.056	0.152
MUFA (% of the total calorie intake)	1st	0.286 *	0.362 **				0.224	−0.008	0.255 *
	2nd	0.313 **	0.189	−0.304 *	−0.230	−0.304 *	0.422 **	0.488 **	0.340 **
PUFA (% of the total calorie intake)	1st	0.106	0.182				0.034	−0.070	0.071
	2nd	0.212	0.082	−0.077	−0.063	−0.077	0.284 *	0.349 **	0.201
LA (% of the total calorie intake)	1st	0.094	0.182				0.013	−0.119	0.056
	2nd	0.212	0.084	−0.072	−0.060	−0.072	0.272 *	0.315 **	0.191
ALA (% of the total calorie intake)	1st	0.009	0.034				−0.028	0.088	−0.018
	2nd	0.143	0.061	−0.100	−0.078	−0.100	0.238 *	0.352 **	0.186
EPA + DHA (mg)	1st	−0.135	0.045				0.026	0.050	0.125
	2nd	−0.043	−0.062	0.058	0.047	0.058	−0.001	0.119	0.010
Carbohydrates (g)	1st	0.247 *	0.066				0.195	0.209	0.049
	2nd	0.132	0.124	−0.108	−0.036	−0.108	0.115	0.057	0.101
Sucrose (g)	1st	−0.015	−0.079				−0.126	−0.016	−0.183
	2nd	−0.042	0.059	−0.046	0.056	−0.046	−0.014	−0.103	0.055
Sucrose (% of the total calorie intake)	1st	−0.245 *	−0.229				−0.335 **	−0.129	−0.322 **
	2nd	−0.177	−0.067	0.117	0.225	0.117	−0.200	−0.287 *	−0.123
Dietary fiber (g)	1st	0.303 *	0.202				0.360 **	0.146	0.278 *
	2nd	-0.024	−0.060	0.039	−0.043	0.039	−0.060	−0.022	−0.092
Carbohydrates (% of the total calorie intake)	1st	−0.261 *	−0.306 **				−0.266 *	−0.014	−0.289 *
	2nd	−0.195	−0.098	0.247 *	0.332 **	0.247 *	−0.333 **	−0.438 **	−0.277 *
Cholesterol (mg)	1st	0.448 **	0.309 **				0.551 **	0.304 *	0.424 **
	2nd	0.187	0.091	−0183	−0.150	−0.183	0.292	0.396	0.230

1st—1st stage of the study (preoperative, baseline); 2nd—2nd stage of the study (12 months after the surgery); * *p* < 0.05; ** *p* < 0.01.

**Table 8:** Correlations between energy, selected nutrient intake, and biochemical parameters across study duration.

Energy/Nutrients	Stage of the Study	Biochemical Parameters				
		FBG (mg/dL)	TC (mg/dL)	TG (mg/dL)	HDL-C (mg/dL)	LDL-C (mg/dL)
Energy (kcal)	1st	−0.061	0.033	0.047	−0.309 **	0.112
	2nd	0.152	0.079	0.367 **	0.011	−0.033
Proteins (g)	1st	0.029	0.038	0.025	−0.134	0.076
	2nd	0.135	−0.049	−0.015	0.178	−0.137
Proteins (% of the total calorie intake)	1st	0.115	−0.018	−0.008	0.239 *	−0.079
	2nd	−0.019	−0.121	−0.308 **	0.203	−0.122
Fats (% of the total calorie intake)	1st	0.284 *	−0.101	0.029	−0.084	−0.110
	2nd	0.196	0.174	0.279 *	−0.103	0.110
SAFA (% of the total calorie intake)	1st	0.162	−0.101	0.039	0.002	−0.137
	2nd	0.165	0.049	−0.075	0.136	−0.070
MUFA (% of the total calorie intake)	1st	0.232	−0.083	0.032	−0.164	−0.065
	2nd	0.150	0.218	0.401 **	−0.089	0.152
PUFA (% of the total calorie intake)	1st	−0.033	−0.031	−0.040	−0.003	−0.014
	2nd	0.019	0.098	0.172	−0.172	0.197
LA (% of the total calorie intake)	1st	0.010	−0.034	−0.046	−0.002	−0.020
	2nd	0.006	0.053	0.156	−0.171	0.159
ALA (% of the total calorie intake)	1st	−0.107	−0.056	−0.023	−0.023	−0.035
	2nd	0.010	0.302 *	0.228	−0.124	0.329 **
EPA + DHA (mg)	1st	−0.219	−0.065	−0.096	0.200	−0.039
	2nd	0.053	0.032	−0.056	0.042	0.042
Carbohydrates (g)	1st	−0.129	0.048	−0.006	−0.266 *	0.142
	2nd	−0.013	0.030	0.272 *	−0.059	−0.010
Sucrose (g)	1st	−0.349 **	−0.034	−0.010	−0.158	0.047
	2nd	−0.064	0.135	0.173	0.002	0.044
Sucrose (% of the total calorie intake)	1st	−0.393 **	0.006	−0.009	−0.046	0.049
	2nd	−0.183	0.067	−0.041	0.012	0.036
Dietary fiber (g)	1st	0.032	0.010	−0.066	−0.216	0.073
	2nd	−0.021	0.087	0.023	0.000	0.126
Carbohydrates (% of the total calorie intake)	1st	−0.324 **	0.106	−0.047	0.007	0.139
	2nd	−0.192	−0.100	−0.019	−0.077	−0.022
Cholesterol (mg)	1st	0.193	−0.108	0.071	−0.164	−0.010
	2nd	0.198	0.043	0.152	−0.073	−0.020

1st—1st stage of the study (preoperative, baseline); 2nd—2nd stage of the study (12 months after the surgery); * *p* < 0.05; ** *p* < 0.01.

## Data Availability

The data presented in this study are available upon request from the corresponding author. The data are not publicly available as they are still being used for analysis and manuscript writing.
